# A systematic review of attention deficit hyperactivity disorder (ADHD) and mathematical ability: current findings and future implications

**DOI:** 10.1186/s12916-015-0414-4

**Published:** 2015-08-27

**Authors:** Maria Grazia Tosto, Sukhleen Kaur Momi, Philip Asherson, Karim Malki

**Affiliations:** King’s College London, MRC Social, Genetic and Developmental Psychiatry Centre (SGDP), Institute of Psychiatry, Psychology & Neuroscience (IoPPN), (PO80), De Crespigny Park, Denmark Hill, London, SE5 8AF UK; Laboratory for Cognitive Investigations and Behavioural Genetics, Tomsk State University, Tomsk, Russia

**Keywords:** Attention deficit hyperactivity disorder, Hyperkinetic disorder, Mathematical ability, Mathematics achievement

## Abstract

**Background:**

Several recent behavioural and behavioural genetic studies have investigated the relationship between attention deficit hyperactivity disorder (ADHD) and mathematical ability. The aim of this systematic review was to provide an overview of these studies to date. An emphasis was placed on reviewing results that explored the association between mathematics and the two ADHD components of attention and hyperactivity-impulsivity separately.

**Methods:**

A systematic search of quantitative studies investigating the association between mathematics and ADHD was conducted across five databases (PsychINFO, Web of Science, PubMed, EMBASE, and Scopus). A total of 30 cross-sectional and four longitudinal studies were included in this review.

**Results:**

Narrative synthesis of the results was provided using PRISMA guidelines. Taken together, the studies pointed at substantial evidence for a negative association between ADHD symptoms and mathematical ability. This association was particularly marked for the inattentive component of ADHD than for the hyperactive-impulsive component. Evidence from twin studies also showed a significant genetic correlation between mathematics and ADHD, which was greater for the inattentive component of ADHD compared to the hyperactive-impulsive component.

**Conclusions:**

The differential relationship of the hyperactivity-impulsivity and inattention domains with mathematics emphasises the heterogeneity within the disorder and suggests a partially different aetiology of the two ADHD domains. A better understanding of the aetiology of ADHD could help develop more efficient interventions aimed at the reduction of its symptoms. It could also offer an explanatory framework for shortcomings in achievement and inform the development of non-pharmacological intervention strategies.

**Electronic supplementary material:**

The online version of this article (doi:10.1186/s12916-015-0414-4) contains supplementary material, which is available to authorized users.

## Background

### Rationale

Attention deficit hyperactivity disorder (ADHD) is a neurodevelopmental disorder with a high global prevalence of 5.29 % [1], characterised by severe and impairing difficulties with sustained attention, restless overactivity, and impulse control. The International Classification of Diseases, 10^th^ edition (ICD-10) and The Diagnostic and Statistical Manual of Mental Disorders, fourth edition (DSM-IV, recently updated to DSM-V) are widely used to diagnose children, adolescents, and adults with ADHD. Both manuals use a diagnostic criteria consisting of the same 18 symptoms and identify the same two core domains reflecting inattentive and hyperactive-impulsive behaviours. The symptoms are persistent across development and have significant effects in adaptive functioning. The DSM criteria identify a broader range of individuals, including those presenting with predominantly inattentive symptoms and an older age of onset. Depending on the features that are most prominent, three clinical presentations of ADHD are described: predominantly inattentive (ADHD-I), predominantly hyperactive-impulsive (ADHD-H), and combined (ADHD-C) type clinical presentations [2].

Although inattention, hyperactivity, and impulsivity represent core symptoms of ADHD, the disorder is highly heterogeneous and is associated with a plethora of different impairments including cognitive and behavioural deficits. Students with ADHD generally show poor academic outcomes relative to their general cognitive abilities, with greater grade repetitions and increased school dropout rates [3, 4]. Due to the complex nature of ADHD, much remains to be understood about the processes underlying the observed educational difficulties. Nevertheless, children diagnosed with ADHD are often educated in general classrooms together with children without the disorder [5].

Mathematical training makes up a large portion of school education and it is positively correlated with longer educational duration and higher qualification attainment [6]. Furthermore, mathematical ability has been shown to be positively associated with socio-economic status in adulthood due to more opportunities in post-secondary education and career development [7].

Much of the existing literature has focused on the links between ADHD and reading disability [8–10], neglecting the important association with mathematics. However, recent studies have demonstrated links between mathematical ability and ADHD. Several studies have linked attentional processes in ADHD with mathematical abilities [11, 12]. As attention plays a key role in mathematical ability, investigations focusing on the relationship between the inattentive domain of ADHD and mathematics may provide further insight into the disorder and the mechanisms of mathematical learning.

Reviews relating to mathematical abilities and ADHD have been sparse in the past due to the limited number of studies focusing on mathematics and ADHD. In this study, we have used the Preferred Reporting Items for Systematic Reviews and Meta-Analyses (PRISMA) guidelines [13] to present a systematic review of empirical evidence currently available on mathematical abilities in individuals diagnosed with ADHD.

### Objectives

We examined existing literature in order to appraise the co-occurrence of mathematical problems and ADHD in all age groups and to explore whether mathematics has a differential relationship with each of the two components of ADHD, namely inattentiveness and hyperactivity-impulsivity.

## Methods

### Protocol and registration

The protocol of this review has been registered with the International Prospective Register of Systematic Reviews (PROSPERO; http://www.crd.york.ac.uk/prospero/, reference CRD42015016186).

### Eligibility criteria

Inclusion criteria consisted of (1) articles published in English in peer-reviewed journals; (2) usage of appropriate empirical research methods, assessed using the quality appraisal criteria (see quality assessment section); (3) cases of any age who met the ADHD criteria (DSM-defined ADHD or ICD-defined hyperkinetic disorder) and/or were assessed for symptoms of the disorder on ADHD validated scales (e.g. Conners [14]); (4) standardized and validated tests assessing mathematical performance rather than school achievement; this allowed us to minimise biases in the assessment of mathematical ability introduced by discrepancy in curricula and school programmes; (5) studies where cases were selected for other learning disabilities in addition to ADHD were excluded (given the comorbidity between mathematical problems and learning disability [15, 16] this further exclusion ensures that reported discrepancies in mathematics performance between the cases and controls are driven by ADHD rather than other comorbid learning disabilities); (6) where the study uses a control group, this should include only healthy individuals, with no symptoms of psychiatric or learning disability; (7) further exclusion was extended to studies with primary aims to investigate cognitive function in relation to ADHD if the correlation between mathematical ability and ADHD was not tested or described (it is important to note that there is no gold standard to define mathematical ability; mathematics is a continuously distributed trait where ability and disability are identified using arbitrary cut-offs along a continua of performance); (8) for this reason, this article reviews studies where individuals diagnosed with ADHD are not solely selected for mathematical disabilities; (9) investigations primarily evaluating the effects of pharmacological or non-pharmacological interventions on mathematical ability of children with ADHD were also excluded; and (10) studies that did not take medication into account and studies that adjusted the data analysis for usage of psychostimulant medication or asked the participants to stop medication 24–48 hours prior to testing mathematical ability were included.

### Information sources

Five databases, including PsychINFO, EMBASE 1806, Web of Science, PubMed, and were searched for articles. An additional manual search of the literature was performed to identify any publications missed by the database search.

### Search

The search was conducted on 1 February, 2015 without any constraints on the year of publication to allow for a thorough and complete review of the literature using the following key words: “attention deficit hyperactivity disorder” OR “ADHD” OR “hyperkinetic disorder” AND “mathematical ability” OR “math* achievement” OR “acalculia” OR “mathematics”.

### Study selection

**Fig. 1:**
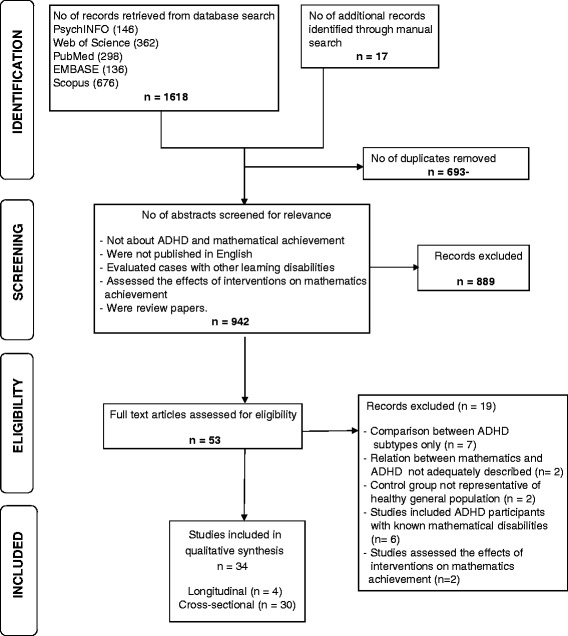
Flow chart of the systematic search and review process conducted in accordance with PRISMA (Preferred Reporting Items for Systematic Reviews and Meta-Analyses) statement criteria

The study selection process is illustrated in Fig. 1. In the first stage, any duplicates were removed and abstracts were subsequently assessed for their relevance in accordance to the inclusion/exclusion criteria. Papers were rejected if they (1) were clearly not about ADHD and mathematical (or mentioned academic) achievement; (2) were not published in the English language; (3) evaluated cases with other learning disabilities along with ADHD; (4) assessed the effects of interventions on mathematics achievement; or (5) were review papers.

Full documents were obtained for the remaining records and checked against the eligibility criteria. Papers were rejected at this stage if (1) no healthy control group was included for comparison (e.g. comparison in mathematics was performed between individuals with ADHD and individuals with other learning disabilities for studies that included a control group); or (2) individuals with ADHD also had known mathematical disabilities. The papers that met the eligibility requirements, based on the above screening criteria, are summarized in Table 1.

**Table 1:** Results of the quality assessment of studies

Domain criterion		1	2	3	4	5	6	7	8	9	10	11	Score	Rating
1	Antonini et al. [35]	+	+	+	+	n/a	n/a	+	+	−	+	+	8	High
2	August et al. [49]	+	+	−	+	n/a	n/a	+	+	+	+	+	8	High
3	Barry et al. [32]	+	−	+	+	n/a	n/a	+	+	+	+	+	8	High
4	Bauermeister et al. [31]	+	+	+	+	n/a	n/a	+	+	+	+	+	9	High
5	Benedetto-Nasho & Tannock [25]	+	+	+	−	n/a	n/a	+	–	+	+	+	7	High
6	Biederman et al. [44]	+	+	+	+	n/a	n/a	+	+	–	+	+	8	High
7*	Biederman et al. [37]	+	+	+	+	+	+	+	+	+	−	+	10	High
8*	Biederman et al. [46]	+	+	+	+	+	+	+	+	+	−	+	10	High
9	Biederman et al. [40]	+	+	+	+	n/a	n/a	+	+	+	−	+	8	High
10	DuPaul et al. [5]	+	+	+	−	n/a	n/a	+	+	−	+	+	7	High
11	Efron et al. [41]	+	+	+	+	n/a	n/a	+	+	−	+	+	9	High
12*	Faraone et al. [18]	+	+	+	−	+	+	−	+	−	−	+	7	Medium
13	Faraone et al. [45]	+	+	–	+	n/a	n/a	+	+	–	+	+	7	High
14	Frick et al. [26]	+	+	+	–	n/a	n/a	–	–	+	+	–	5	Medium
15	Gremillion & Martel [36]	+	+	+	+	n/a	n/a	+	+	+	+	+	9	High
16	Greven et al. [50]	+	+	+	+	n/a	n/a	–	+	+	+	+	8	High
17	Hart et al. [19]	+	+	+	–	n/a	n/a	–	+	+	+	+	7	High
18	Kaufmann & Nuerk [20]	+	+	–	–	n/a	n/a	+	–	–	+	+	5	Medium
19	Kempton et al. [28]	+	+	+	–	n/a	n/a	+	+	+	–	+	7	High
20	Laasonen et al. [33]	+	+	+	+	n/a	n/a	–	+	–	+	–	6	Medium
21	Lamminmäki et al. [39]	+	+	–	+	n/a	n/a	+	+	–	+	+	7	High
22	Lewandowski et al. [21]	+	+	+	–	n/a	n/a	+	+	–	+	+	7	High
23*	Massetti et al. [38]	+	+	–	+	+	+	+	+	–	+	+	9	High
24	Mayes & Calhoun [42]	+	+	–	+	n/a	n/a	–	+	+	–	+	6	Medium
25	Mealer et al. [22]	+	+	–	–	n/a	n/a	+	+	–	+	+	6	Medium
26	Papaioannou et al. [48]	+	+	+	+	n/a	n/a	+	+	–	+	+	7	High
27	Penny et al. [29]	+	+	–	–	n/a	n/a	+	+	–	+	+	6	Medium
28	Roy-Byrne et al. [34]	+	+	+	+	n/a	n/a	+	+	+	+	+	9	High
29	Rucklidge & Tannock [23]	+	+	–	–	n/a	n/a	+	+	+	+	+	7	High
30	Schachar & Tannock [30]	+	+	+	–	n/a	n/a	–	+	–	+	+	6	Medium
31	Seidman et al. [24]	+	–	+	–	n/a	n/a	+	+	–	+	+	6	Medium
32	Thorell [27]	+	+	+	–	n/a	n/a	–	–	+	+	+	6	Medium
33	Todd et al. [43]	+	+	+	+	n/a	n/a	+	+	+	+	+	9	High
34	Zentall et al. [47]	+	+	+	+	n/a	n/a	+	+	+	+	+	9	High

n/a, Not applicable; Domain criterion, the 11-question criteria used for quality appraisal; +, criteria fulfilled; –, criteria not fulfilled; *, Longitudinal studies; Scores for longitudinal studies: high quality >9, medium quality 5–8, low quality 0–4; Scores for cross-sectional studies: high quality >7, medium quality 4–6, low quality 0–3. Only the four longitudinal studies, indicated with the asterisk (*) fulfil the criteria in columns 5 and 6 of participant response rate and reason for participant drop-out

### Data collection process

Data were extracted by one of the authors and confirmed by a second author; in case of disagreement, a third author was consulted. Extraction was limited to published data; authors of the papers were not contacted for missing information and this is indicated as ‘not reported’ (NR).

### Data items

The following variables were assessed: (1) type of study (experimental, cross-sectional, or longitudinal); (2) nature of the cases (diagnosis of ADHD in absence of learning disabilities); (3) numbers of cases (ADHD) and, if available, number for each of the ADHD sub-groups, inattention and hyperactivity-impulsivity; (4) nature of the control group (healthy controls) and number of healthy controls where a control group was present; (5) age range and sex by group(s); (6) the response rate to the study; (7) dropout rates for participants recruited (if longitudinal study); (8) whether the potential confounding demographic and cognitive factors (IQ, medication (unless temporarily stopped), socio-economic status, and gender) were accounted for in the design and/or in their analysis; (9) whether cases were on medication during the test or whether the medication was stopped temporarily prior to testing; (10) name of the mathematics test used to test mathematical performance; (11) mean mathematics score with standard deviation for cases and controls and/or other appropriate means of describing the association between ADHD and mathematics (e.g. regression, correlation coefficients); (12) if longitudinal study scores were available at baseline and follow-up; and (13) whether effects size were included.

### Risk of bias in individual studies

Risk of bias of individual studies was critically assessed at the study level and taken into account in the quality assessment. The quality assessment of the mixed methods studies reviewed was performed using items provided in the Critical Appraisal Skills Programme, Oxford checklist tool [17] (see Additional file 1 for items list).

### Summary measures

Samples descriptive statistics include age range, mean, and standard deviation of age by group, together with total number of cases and controls and whether cases were medicated or not (Table 2). The table further summarizes means and standard deviation of mathematics, either in raw or standardized scores (z-scores) and the name of the test used to assess mathematical performance. The association between ADHD and mathematics has been evaluated by mean differences (*t*-test), regression coefficients (β), correlation coefficient (*r*), z-scores, genetic, shared and non-shared environmental correlations (r_a_, r_c_, and r_e_, respectively), or genetic covariances. The statistical significance (*P* value) for the results of each analysis was also included in the summary table.

**Table 2:** Descriptive statistics of samples and of mathematical performance

	Participants descriptive statistics by group				Mathematical test descriptive statistics				
Publication	Age range whole sample	N (mean age, SD age)		On medication during test	Test name	N (mean score, SD score)		*P* value	Quality rating
		*ADHD group*	*Non-ADHD group*			*ADHD group*	*Non-ADHD group*		
Antonini et al. [35]	7–11	49 (7.92, 1.11)^C^	45 (8.29, 1.34)	No	WIAT-II	97.06 (13.19)^C^	112.84 (18.7)	**<0.0001** ^**C**^	High
		53 (8.36, 1.30)^I^				97.89 (13.79)^I^		**<0.0001** ^**I**^	
August et al. [49]	6–11	79 (8.90, 1.17)	61 (8.70, 1.19)	NR	WRAT-R	92.80 (16)	93.5 (17)	>0.05	High
Barry et al. [32]	8–14	30 (11.10, 1.30)^C^	33 (11.24, 1.20)	No	MBA	1.52 (12.38)^a^	10.39 (12.77)^a^	**<0.05**	High
		3 (11.10, 1.30)^I^							
Bauermeister et al. [31]	6–11	140 [(32.1%)^C^; (17.1%)^I^; (2.8%)^U^] (8.31, 1.70)	NR (8.31, 1.70)	No	WPB-S	β = −0.33^I^		**<0.001**	High
Benedetto-Nasho et al. [25]	7–11	14 (9.55, 1.01)	15 (9.02, 1.14)	No	MCW	21.66 (12.13)^P^	47.32 (15.20)^P^	**<0.001** ^**P**^	High
						52.53 (35.79)^A^	76.98 (18.04)^A^	**<0.05** ^**A**^	
						11.31 (10.92)^E^	36.79 (15.24)^E^	**<0.001** ^**E**^	
Biederman et al. [44]	Adults	84 (38.90, 9)	142 (NR)	Yes	WRAT-R	101.8 (15.30)	108.5 (14.70)	**<0.001**	High
Biederman et al. [37]	6–17	128 (10.60, 3.00)^b^	109 (11.60, 3.70)^b^	Yes	WRAT-R	96.8 (17.40)^b^	111.30 (16.10)^b^	**<** **0.05** ^**b**^	High
		128 (14.40, 3.10)^f^	109 (15.2, 3.70)^f^			93.4 (18.30)^f^	109.50 (15.70)^f^	**<** **0.05** ^**f**^	
Biederman et al. [46]	6–17	100 (9.0, NR)^Y^	69 (8.90, NR)^Y^	NR	WRAT-R	96.70 (16.50)^Y,b^	111.70 (15.90)^Y,b^	**<0.001**	High
						93.30 (18.30)^Y,f^	110.10 (16.20)^Y,f^		
		40 (14.40, NR)^O^	51 (15.20, NR)^O^			96.80 (19.00)^O,b^	111.10 (17.10)^O,b^		
						93.80 (18.20)^O,f^	108.40 (14.80)^O,f^		
Biederman et al. [40]	6–18	140 (11.20, 3.40)	122 (12.20,	Yes	WRAT	95.50 (13.30)	106.20 (15.40)	**<0.001**	High
DuPaul et al. [5]	NR	95 (8.50, 1.20)^C^	53 (8.50, 1.10)	Yes	WJ-III	94.50 (12.70)	113.40 (10.30)	**<0.001**	High
		31 (8.50, 1.20)^I^							
		10 (8.50, 1.20)^H^							
Efron et al. [41]	6 – 8	93 (7.3, 0.4)^C^	212 (7.3, 0.4)	Yes	WRAT	90.2 (14.7)	102.8 (13.4)	**<0.001**	High
		64 (7.3, 0.4)^I^						Subtypes = NS individually	
		22 (7.3, 0.4)^H^				β = –0.69			
Faraone et al. [18]	6–17	93 (10.7, 3.10)^b^	120 (11.6, 3.70)^b^	Yes	WRAT-R	103.60 (14.40)^b^	111.30 (16.10)^b^	**<0.05** ^b^	Medium
		83 (14.8, 3.20)^f^	109 (15.5, 3.70)^f^			98.80 (16.10)^f^	109.50 (15.70)^f^	**<0.05** ^f^	
Faraone et al. [45]	6–17	485 (10.90, NR)^Y^	78 (10.80, NR)^Y^	NR	WRAT-R	92.70 (22.50)^Y^	110 (15.20)^Y^	**<0.001**	High
		326 (10.90, NR)^O^	54 (10.80, NR)^O^			88 (25.00)^O^	111.8 (15.7)^O^		
Frick et al. [26]	7–12	92 (9.50, NR)^C^	42 (10.60, NR)	No	BASIS	Underachievers: 14 % ^C^, 7 % ^I^	Underachievers:2%	**<0.05** ^**C**^	Medium
		13 (9.50, NR)^I^						>0.05^I^	
Gremillion et al. [36]	6–12	266 (9.72, 1.50)	207 (9.79, 1.48)	No	WIAT-II	40.12 (9.39)	43.09 (9.22)	**<0.001**	
						r = −0.27^C^		**<0.001**	High
						r = −0.22^I^		**<0.001**	
						r = −0.28^H^		**<0.001**	
Greven et al. [50]	NR	2191 (12, NR)^MZ^		NR	UNT, NNPT,CKT	Genetic correlation:			High
		3930 (12, NR)^DZ^				r_a_ = –0.41 (95 % CI: –0.47, –0.37)^I^		**Significant**	
						r_c_ = 0.12 (95 % CI: –0.12, 0.35)^I^		**NS**	
						r_e_ = –0.20 (95 % CI: –0.23, –0.16)^I^		**Significant**	
						r_a_ = –0.22 (95 % CI: –0.28, –0.17)^H^		**Significant**	
						r_c_ = –0.27 (95 % CI: –0.44, –0.04)^H^		**Significant**	
						r_e_ = 0.00 (95 % CI: –0.04, 0.04)^H^		**NS**	
						Phenotypic correlation:			
						r = –0.26 (95 % CI: –0.28, –0.24)^I^		**Significant**	
						r = –0.18 (95 % CI: –0.20, –0.16)^H^		**Significant**	
Hart et al. [19]	NR	271 (9.82, 0.99)^MZ^		NR	WJ-III	Genetic Covariance:		**<0.05** ^**I**^	High
		159 (9.82, 0.99)^DZ^				covar = 0.36 (95 % CI: 0.23, 0.50)^I^			
						covar = 0.31 (95 % CI: 0.27, 0.43)^H^			
						Shared environment:		**<0.05** ^**H**^	
						covar = 0.90 (95 % CI: 0.84, 0.92)^I^			
						covar = 0.90 (95 % CI: 0.89, 0.92)^H^			
Kaufmann & Nuerk [20]	8.8 –11.7	16 (10.20, 1.40)	16 (10.40, 1.30)	NR	NV-CNR	91.4 (10.16)	97.27 (2.55)	**0.02**	Medium
						75 (24.15)	81.25 (17.08)		
					V-CNR	96.15 (4.58)	98.44 (2.54)	0.45	
						97.92 (3.02)	95.83 (11.39)		
						99.06 (2.02)	99.69 (1.25)		
					SMC	94.8 (5.61)	96.68 (3.12)	0.65	
					CMC	56.26 (20.20)	68.36 (18.45)	0.30	
					WMC	70.84 (25.97)	79.16 (17.87)	0.50	
Kempton et al. [28]	6–12	15 (8.65, 1.53)	15 (8.81, 1.48)	No	WRAT	83.00 (10.92)	88.6 (15.72)	>0.05	High
Laasonen et al. [33]	18–55	30 (31.60, 8.17)	40 (37.15, 11.70)	No	WAIS-III	10.90 (3.17)	12.18 (2.40)	>0.05	Medium
Lamminmäki et al. [39]	NR	17 (8.67, 1.28)^C^	22 (8.92, 1.4)	Yes	WJ-III	Z-score:	–0.53 (0.83)	0.070	High
						–1.33 (0.06)^C^			
		20 (9.78, 1.33)^I^				–0.88 (0.96)^I^			
		8 (8.60, 1.29)^H^				–0.50 (1.14)^H^			
Lewandowski et al. [21]	10–13	17 (11, NR)^C^	27 (11, NR)	Yes	WJ-III	92.59 (14.71)	102.11 (13.42)	**<0.05**	High
		7 (11, NR)^I^							
		3 (11, NR)^H^							
Massetti et al. [38]	4–6^b^	85 (5.20, 0.70)^C^	130 (5.20, 0.08)	Yes	WJ-III	β = −2.55, z = −1.92^C^ *(refers to longitudinal performance of children exhibiting ADHD-C symptoms across all waves of assessment)*		0.060^C^	High
		14 (5.70, 0.50)^I^				β = −6.49, z = −3.34^I^ *(refers to overall longitudinal performance)*		**<0.001** ^**I**^	
	12–14 ^f^	26 (5.10, 0.80)^H^				β = 0.40, z = 0.18^H^ *(refers to overall longitudinal performance*)		0.360^H^	
						β = −7.27, z = −3.61^C,f^ *(overall performance using DSM-IV number of impairment settings to define subtypes)*		**<0.001** ^**C,f**^	
						ADHD-I & ADHD-H^f^ = NS		>0.05	
Mayes & Calhoun [42]	6–16	724 (9, 2)	149 (9, 2)	Yes	WIAT, WIAT-II, WRAT-III	9 %^d^	4 %^d^	**<0.001**	Medium
Mealer et al. [22]	6–13	20 (8.90, 2.08)	20 (8.50, 1.93)	No	WISC - III	8.70 (3.52)	10.60 (2.7)	0.063	Medium
Papaioannou et al. [48]	6–11		835 (103.90, 17.60)	NR	STAA	Z-score:	Z-score:	**0.003** ^**C**^	High
		24 (109.30, 17.10)^C^				−0.80 (1.11)^C^	08 (0.97)		
		31 (103.90, 16.40)^H^				−0.25 (0.99)^H^		>0.5^H^	
		33 (100.10, 16.40)^I^				−0.78 (1.07)^I^		**0.0001** ^**I**^	
Penny et al. [29]	6–12	32 (8.65, 1.48)^C^	19 (8.40, 1.40)	No	WRAT-III	82.20 (17)	89.80 (15)	>0.05	Medium
		1^I^							
Roy-Byrne et al. [34]	18–64	46 (33.10, 9.70)	46 (39.50, 11.20)	No	WRAT-R	90.20 (19.90)	100.60 (23.90)	>0.05	High
Rucklidge et al. [23]	13–16	24 (14.68, 1.51)^F^	28 (15.31, 1.04)^F^	No	WRAT-III	96.33 (13.85)^F^	112.78 (12.34)^F^	**<0.001**	High
		35 (14.80, 1.22) ^M^	20 (14.8, 1.22)^M^			90.57 (15.697)^M^	108.20 (10.11)^M^	NR	
Schachar & Tannock [30]	7–11	22 (9.20, 1.20)	16 (9.0, 1.4)	NR	WRAT-R	92.40 (9.00)	97.60 (13.80)	>0.05	Medium
Seidman et al. [24]	6–17	43 (NR, NR)	36 (NR, NR)	Yes	WRAT-R	95.70 (16.00)	107.60 (14.30)	**<0.05**	Medium
Thorell [27]	6–7	21 (6.30, 0.49)	124 (6.30, 0.49)	NR	NS	r = –0.28^I^		**<0.001** ^**I**^	Medium
						r = –0.13^H^		>0.05^H^	
Todd et al. [43]	7–17	149 (13.70, 3.00)^C^	731 (14.20, 3.10)	Yes	WRAT-III	87.3 (13.60)^C^		**<0.001** ^**C**^	High
		243 (14.30, 3.00)^I^				89.40 (13.50)^I^	96.60 (13.40)	**<0.001** ^**I**^	
		31 (15.30, 3.10)^H^				95.50 (11.20)^H^		>0.05^H^	
Zentall et al. [47]	7–15	107 (NR, NR)	121 (NR, NR)	NR	CAT	55.64 (2.97)	75.11 (3.00)	**<0.001**	High
					TAT	F(2,223) = 58.5 *(addition)*	61.23 (NR)	**<0.001**	
						F(2,223) = 27.95 *(subtraction)*	32.60 (NR)		
						F(2,205) = 75.23 *(multiplication)*	91.79 (NR)		

Descriptives of samples and of mathematical performance. Numbers in bold highlight significant results

N, Sample size; SD, Standard deviation; NR, Not reported; 95 % CI, 95 % confidence interval; Z-score, Represents achievement scores normalized and residualized for intelligence scores; r_a_, Genetic correlation; r_c_, Shared environmental correlation; r_e_, Non-shared environmental correlation; covar, Covariance; ^C^, Predominantly combined-type; ^I^, Predominantly inattentive-type; ^H^, Predominantly hyperactive-type; ^U^, Unspecified; ^MZ^, Monozygotic twin pairs; ^DZ^, Dizygotic twin pairs; ^Y^, Young; ^O^, Old (adolescents); ^b^, Baseline; ^f^, Follow-up; ^F^, Female; ^M^, Male; ^a^, The values demonstrate discrepancy between predicted achievement on the Kaufman Brief Intelligence Test Composite [58] and actual math achievement on the Mini Battery of Achievement (MBA) test [59] with a positive score representing actual achievement above the predicted score as estimated by participant’s intellectual level, and a negative score representing actual achievement below the predicted score; ^P^, Productivity (number of math problems attempted out of the total); ^A^, Accuracy (percentage of problems answered correctly out of those attempted); ^E^, Efficiency (number of correctly completed items out of total number of items available); ^d^, Discrepancy between Intelligence quotient (IQ) and mathematics score

NB: on average, ADHD children scored lower on the test than their intended IQ (<IQ of 104.8) and controls scored higher on the test than their intended IQ (>IQ of 97.5)

Tests: WIAT-II, Wechsler Individual Achievement Test – Second Edition; WRAT-R, Wide Range Achievement Test-Revised; MBA, Woodcock-McGrew-Werder Mini-Battery of Achievement; WPB-S, Woodcock Psychoeducational Battery–Spanish; MCW, Math computational worksheet; WRAT, Wide Range Achievement Test; WJ-III, Woodcock-Johnson III achievement test; BASIS, Basic Achievement Skills Individual Screener; UNT, Understanding Number test; NNPT, Non-numerical Processes test; CKT, Computation and Knowledge test; BA, Basic arithmetic; NV-CNR, Core numerical representations – non-verbal magnitude representations; V-CNR, Core numerical representations – verbal representations; SMC, Simple mental calculation; CMC, Complex mental calculation; WMC, Written mental calculation; WAIS-III, Wechsler Adult Intelligence Scale – Third edition; WJ-R, Woodcock-Johnson achievement test-Revised; WIAT-NO, Wechsler Individual Achievement Test Numerical Operations; WIAT, Wechsler Individual Achievement Test; WRAT-III, Wide Range Achievement Test-Third Edition; WISC-III, Wechsler Intelligence Scale for Children – Third Edition; STAA , Screening test of arithmetic ability; PMS, Pupil Monitoring System; NS, Not specified; CAT, California Achievement Test; TAT, Timed Arithmetic Trial

### Synthesis of results

A narrative synthesis of the findings from the studies was performed and reported following PRISMA guidelines.

## Results

### Study selection

The number of studies emerging from the selection process is illustrated in Fig. 1.

### Study characteristics

Cross-sectional and longitudinal (marked by (*) in Table 1) studies included in this review assessed the difference between ADHD cases and healthy controls and/or the association between ADHD (combined or by subtype) and mathematical performance. The characteristics of cases and controls, the nature of comparison, and outcomes are summarised in Table 2.

### Risk of bias within studies

Data on the risk of bias and quality assessment for each study are presented in Table 1. Fourteen studies had relatively small sample sizes (item number 4 on the quality assessment) [5, 18–30]; eight of these studies commented on the small sample size or reported a small effect size with low statistical power [5, 18–24]. Overall, 12 studies reported their cases as not having any drug treatment for the disorder [22, 23, 25, 26, 28, 29, 31–36]; 12 reported cases using medications [5, 18, 21, 24, 37–44]; and 10 did not give any information about the cases’ treatment status [19, 20, 27, 30, 45–50]. Out of the 22 studies that used medicated cases or did not report medication status, 86 % reported an association between mathematics and ADHD (19 studies: [5, 18–21, 24, 27, 37, 38, 40–48, 50]). Only 58 % of those that did not include medicated cases reported a significant association (7 studies: [23, 25, 26, 31, 32, 35, 36]). Each study had its own set of limitations (Table 1), most studies matched cases and control on variables such as age and sex and had fairly large sample sizes; some of them controlled for factors such as IQ and socioeconomic status, and one study controlled for medications [38]. For the studies where these variables were not controlled for, data for such variables was not provided; it was unclear whether this lack of information was due to investigators not considering these factors as potential confounders, or due to unwillingness of the participants to disclose information. In order to control for age, two studies compared children and adolescents with ADHD (all DSM-IV subtypes) to matched controls separately [45, 46]. Out of the 34 studies included in this review, 24 were rated as high quality [5, 19, 21, 23, 25, 28, 31, 32, 34–41, 43–50] and 10 as medium [18, 20, 22, 24, 26, 27, 29, 30, 33, 42].

### Results of individual studies

The most common tests used to asses mathematical performance (Table 2) were the following: the Wide Range Achievement Test 3rd-edition/revised [51] used in 16 studies [18, 23, 24, 28–30, 34, 37, 40–46, 49]; the Woodcock-Johnson Test 3rd-edition/revised [52] used in five studies [5, 19, 21, 38, 39], and the Wechsler Individual Achievement Test, 2nd edition/numerical operations [53] used in three studies [35, 36, 42]. Some studies used other mathematical standardised tests (e.g. Wechsler intelligence scale for children -WISC and adults- WAIS-Wechsler Adult Intelligence Scale) [22, 31–33, 47, 48]. The Math Computational Worksheet (MCW) used by Benedetto-Nasho and Tannock [25] and developed by Douglas et al. [54] has not been validated against other standardised tests. Additionally, although the Basic Achievement Skills Individual Screener administered by Frick et al. [26] is designed to quickly screen children’s reading, mathematics, and spelling skills, there is not much empirical evidence in literature for its reliability and validity [55]. Thorell [27] used an unspecified test battery which showed a good test-retest reliability of 0.77 measured using a random sub-sample of 26 children tested two weeks apart, but they did not report its validity. Studies that used other mathematical standardized tests were given a lower score in quality control (Table 1, criteria 8).

Four studies compared mathematical performance at baseline and follow-up [18, 37, 38, 46]. Three studies focused on adolescent/adult samples [33, 34, 44].

Results were evaluated based on the magnitude of the association between mathematics and ADHD and are reported in the sections below using standardised beta coefficients (β) and/or Pearson correlation coefficients (r) and mean difference between cases and controls, where relevant. For genetically sensitive studies, the genetic and environmental correlational coefficients are reported; these are marked as r_a_, r_c_, and r_e_, genetic covariance and shared-environmental covariance.

### The association between ADHD and mathematical ability

Following quality assessment, all papers were rated as either high or medium with no papers achieving a low-quality rating score.

#### High quality rating studies

Out of the 24 studies, 20 reported a statistically significant negative association between ADHD symptoms and mathematical performance [5, 19, 21, 23, 25, 31, 32, 35–38, 40, 41, 43–48, 50]. Among these 20 studies, 11 looked at subtypes, and seven out of these (63%) reported a negative association between mathematics and the inattention subtype [19, 35, 36, 38, 43, 48, 50]. Only three out of these 11 studies (27 %) reported a significant association between hyperactivity-impulsivity and mathematics [19, 36, 50].

Among the three longitudinal studies with high quality ratings, Massetti et al. [38] did not find significant correlation between ADHD-C subtype (n = 85) and mathematical ability (β = −2.55, z = −1.92, *P* = 0.06) during the 8-year period of assessments. However, using a restricted sample of 73 children who exhibited both hyperactive and inattentive symptoms from the first assessment, the association between ADHD-C subtype and mathematics was significant for the same period of time (β = − 7.27, z=−3.61, *P* <0.0005). The other two longitudinal studies [37, 46] showed a negative correlation between ADHD and mathematical performance at baseline and follow-up. Additionally, in children with ADHD, mathematical achievement scores decreased over time. As children in the studies were not selected on mathematical disability, this evidence may suggest a causal link between the disorder and poor school performance later in life.

Rucklidge and Tannock [23] analysed female and male ADHD/control groups separately. They reported females with ADHD to be more impaired in mathematics than female controls (*P* <0.001) but did not report results on any analyses looking at differences between males with ADHD and male controls.

Four studies with high quality ratings did not show any significant correlation between ADHD or its subtypes and mathematical ability [28, 34, 39, 49].

Two behavioural genetic studies used a twin design to explore the genetic (r_a_), shared (r_c_), and non-shared (r_e_) environmental correlations between ADHD and mathematics. Greven et al. [50] found a significant negative phenotypic correlation of mathematical ability and ADHD, with a greater association with inattentive symptoms (r = −0.26; 95 % CI = −0.28, −0.24) than with hyperactive-impulsive symptoms (r = −0.18; 95 % CI = −0.20, −0.16). They also reported a stronger negative genetic correlation of mathematical ability with inattentive symptoms (r_a_ = −0.41; 95 % CI = −0.47, −0.37) compared to hyperactive-impulsive symptoms (r_a_ = −0.22; 95 % CI = −0.28, −0.17), suggesting more genetic factors in common between inattention and mathematics compared to hyperactivity-impulsivity and mathematics. The study reported a small significant shared environmental correlation between mathematics and hyperactivity (r_c_ = –0.27) but a non-significant shared environmental correlation with inattention. This may point to a different aetiology of the covariation between mathematics and the two subtypes. Hart et al. [19] reported a moderate genetic association of mathematics with inattentive symptoms (covariance = 0.36; 95 % CI = 0.23, 0.50) as well as hyperactive-impulsive symptoms (covariance = 0.31; 95 % CI = 0.27, 0.43). However, the study reported a similarly strong shared environmental correlation of mathematics with inattentive symptoms (covariance = 0.90; 95 % CI = 0.84, 0.92) and with hyperactive-impulsive symptoms (covariance = 0.90; 95 % CI = 0.89, 0.92).

#### Medium quality rating studies

Six out of ten medium quality-rating studies reported a significant negative association between ADHD and mathematical performance [18, 20, 24, 26, 27, 42]. Frick et al. [26] reported a significant association only with the ADHD-C group but not with the inattention group. Thorell [27] reported a significant negative correlation between inattentive symptoms and mathematical ability but not with hyperactive-impulsive symptoms. The medium rating, longitudinal study conducted by Faraone et al. [18] reported findings consistent with the other three longitudinal studies [37, 38, 46]. Kaufmann and Nuerk [20] assessed mathematics using five different number processing and calculation tasks but showed a significant impairment only in core numerical representations (non-verbal magnitude representations); participants with ADHD-C found it difficult to compare numbers and determine whether one number was greater or lesser than the other.

The four medium quality rating studies that did not find an association between ADHD and mathematical performance all had a small sample size [22, 29, 30, 33]; in particular, small sample sizes were acknowledged in Mealer et al. [22].

In summary, nine out of the 11 studies looking at the ADHD subtype of inattentive symptoms [19, 27, 31, 35, 36, 38, 43, 48, 50] found a negative correlation between ADHD-I and mathematical performance. Conversely, only three out of the eight studies looking at the hyperactive-impulsive symptoms [19, 36, 50] found an association between ADHD-H and mathematics. Lastly, four out of the six studies looking at the combined symptoms [35, 36, 43, 48] reported a significant association.

## Discussion

### Summary of evidence

This systematic review presents evidence for the negative association between ADHD and mathematical ability. Overall, the majority of the studies (76.47 %) demonstrated a significant association even after controlling for IQ, age, socioeconomic status, and other potential attenuating factors such as psychostimulant medication [38]. Regardless of the statistical power, almost all of the studies reported lower performance on mathematical tests in participants showing ADHD symptoms compared to healthy controls. It is noteworthy that in the studies that investigated the symptom components of ADHD separately, inattentiveness showed a stronger association with mathematical problems compared to the hyperactive-impulsive symptoms (~82 % vs. ~38 %, respectively). Most studies that did not find an association reported a small sample size with the possibility of underpowered analyses. Genetically sensitive studies suggested that the co-occurrence of ADHD and poor mathematical ability is partially explained by common genetic [19, 50] and by common environmental factors [19]. Consistent with the results of phenotypic analyses, these studies also suggested that the genetic link between ADHD and mathematics underperformance may be stronger for the inattentive symptoms of ADHD [50]. In light of these results, it is worth considering the symptomatology of inattentive behaviour and its association with mathematical ability.

During primary school, mathematics education requires increased continuous attention and regular independent seat work, resulting in difficulty in learning for students with ADHD symptoms [5]. In several studies, this difficulty has been attributed to deficits in executive functioning, including planning, organising information, maintaining information for future use, inhibiting an inappropriate response, using working memory, cognitive flexibility, and ability to deduce when provided with limited information [20, 32]. The stronger relationship of attentional factors and mathematics, compared to the hyperactive-impulsive factors, points to a differential relationship of the two ADHD domains with mathematics. This evidence emphasises the heterogeneity of ADHD and points to a partial different aetiology of the two domains. This is further supported by a behavioural genetic study that investigated the direction of the association between inattentiveness and hyperactivity-impulsivity between the ages of 7 and 12 [56]. The study suggested that genetic factors influencing inattentiveness are largely independent from the ones influencing hyperactivity-impulsivity. It also emerged that the association between the two dimensions was largely due to the same genetic factors over time, suggesting that genetic influences contribute to the stability of this association. However, new genetic influences were specific at each age, highlighting the developmental aspect of the disorder. The longitudinal relationship between the two dimensions appeared to be unidirectional, with hyperactivity-impulsivity at the age of 7 predicting the presence of inattentiveness at 12, but not vice versa [56]. This could be interpreted as a causal relationship between the two domains, suggesting that the genetic factors influencing the first domain may have an impact on the second domain via the first.

The ADHD clinical subtypes (as described by the DSM-IV criteria) are developmentally unstable and reflect arbitrary cut offs on dimensional measures of inattention and hyperactivity/impulsivity. Some children exhibiting ADHD-C in early childhood would meet the criteria for ADHD-I later on in childhood or adolescence as the hyperactive symptoms decrease overtime in comparison to the inattentive symptoms [57]. Alternatively, some children who meet the criteria for ADHD-H in early childhood may eventually shift to ADHD-C as their inattentive symptoms become more prominent during the school years [57]. Therefore, when interpreting the results of the various studies, these developmental changes should be kept in mind. This review has identified only four longitudinal studies that explored the association between mathematics and ADHD. Considering the potential impact of development on the disorder, more efforts should be devoted to longitudinal research to gain a better understanding of its aetiology and progression. Findings from these studies could lead to a better classification and help inform non-pharmacological prevention and treatment strategies. We have now entered a new system of classification following the release of DSM-V that allows for comorbid diagnosis of ADHD and Autism and both disorders are associated with impaired mathematical abilities. This review ties together studies that have used diagnostic criteria centred on DSM-IV. From this point forward, studies may use this new way of diagnosis and grouping of cases.

### Limitations

Although this review used the differential relationship of ADHD with mathematics to highlight the heterogeneity within the disorder, there are a number of limitations that need to be considered. Several studies had relatively small sample size and different studies controlled for different variables. Moreover, the comorbidity between ADHD and other conduct disorders and behavioural problems are pervasive and this could not be fully appraised within each study. To control for these factors and evaluate bias we used set items provided in the Critical Appraisal Skills Programme checklist tool [17]. Any tool for evaluating the quality of evidence has its own strengths and limitations. However, we did not use quality ratings as outcome in a quantitative analysis. Therefore, we believe that the appraisal tool can have only limited impact on the overall conclusions of this study which remain compelling. There is no gold standard to define mathematical ability, with various labels or keywords used in the literature to describe mathematical problems. We specifically excluded studies that recruited children with ADHD and mathematical impairment but variations in the arbitrary cut-off thresholds used to determine mathematical disability could alter the significance of comparisons. Furthermore, mathematical ability has been interpreted differently across studies with various tests evaluating different aspects of mathematics. This could have limited the database search, preventing further investigations and insight into the overall results of this review.

Using the association of mathematics with ADHD has allowed highlighting the heterogeneity within the disorder. However, studies exploring the association between mathematics and ADHD are fewer compared to other phenotypes. Therefore, the four longitudinal studies identified in this review did not allow to fully capture the developmental aspect of ADHD.

Our search for papers was restricted to in-print English articles available in online databases only; articles in other languages could have been missed. Finally, the effect of medications could not be fully accounted for.

## Conclusions

Overall, the literature reviewed shows a negative association between ADHD and mathematics and that this association is stronger for the inattentive symptoms; the differential relationship of the two ADHD subtypes with mathematics points to a partially different aetiology within the disorder. Due to varying symptomatology found among children with ADHD, it is important to give an accurate diagnosis according to the two subtypes in order to identify which children are more likely to be at risk of mathematical difficulties. The genetic studies reviewed show that the covariation of ADHD and mathematics is partially due to common genetic factors but environmental factors still play a role. These insights are advantageous to our understanding of this complex disorder and they could help develop non-pharmacological interventions that go beyond simply the reduction of ADHD symptoms. These interventions, used solely or in conjunction with pharmacological treatments, could allow children to deal better with the pressures of the general classroom while having the chance to enjoy and thrive in the educational environment. Further research is needed to investigate specific factors that determine the association between ADHD and deficits in mathematical ability and to show the extent to which they share a common neurobiological basis.

## Additional file

Additional file 1:
**Critical Appraisal Skills Programme, Oxford checklist tool for quality appraisal.** (DOCX 17 kb)

## Abbreviations

ADHD: Attention deficit hyperactivity disorder
DSM-IV: Diagnostic and Statistical Manual of Mental Disorders, fourth edition
PRISMA: Preferred Reporting Items for Systematic Reviews and Meta-Analyses
