# Supplementary material for: A systematic review of attention deficit hyperactivity disorder (ADHD) and mathematical ability: current findings and future implications
Source: BMC Med. 2015 Aug 27;13:204. doi: 10.1186/s12916-015-0414-4 (PMC4552374; doi:10.1186/s12916-015-0414-4)
Supplement: Additional file 1: — Critical Appraisal Skills Programme, Oxford checklist tool for quality appraisal. (DOCX 17 kb) [file 12916_2015_414_MOESM1_ESM.docx]

SUPPLEMENTARY

***Critical Appraisal Skills Programme, Oxford checklist tool for quality appraisal***

The following aspects of the studies were assessed to determine their quality:

*Objective of the study*

1) The study addresses the relationship between ADHD and mathematical ability.

*Participants' selection*

2) The cases recruited met ADHD symptoms according to DSM-based or ICD-based criteria and the eligibility criteria.

3) The controls were selected using appropriate screening methods to minimise bias and were reflective of the general healthy population (e.g. if some studies recruited controls from general classrooms but others recruited opportunity samples through advertisement placed in hospitals. this could have resulted in a more heterogeneous control group that may present other psychiatric disorders associated with mathematical impairments)

4) The study selected sufficient number of cases and controls. This was evaluated by objective information (i.e. effect size and significance of results) together with authors' report on the power of the sample size.

*5) The response rate to the study was sufficiently high (>40%).

*6) There was adequate and clear description of the participant dropout rate and the reasons for dropouts.

NB: The criteria with the asterisk (*) were only applied to longitudinal studies as the response rate and dropout rate are not relevant in determining the validity of methodology and findings of a cross-sectional study.

*Methodology and outcome measurements*

7) The demographics of the sample were clearly defined (including age range and sex).

8) The authors used standardized and validated tools to measure mathematical ability.

9) The authors accounted for potential confounding demographic and cognitive factors (IQ, medication - unless temporarily stopped, socio-economic status and gender) in the design and/or in their analysis.

10) During the assessments, both control and non-control participants were given identical instructions. The tests were performed under controlled conditions. For example, the mathematical tests were administered in a quiet room without any distractions (as this could be a potential confounding factor) and appropriate time constraints (if needed).

11) Appropriate methods were used to analyse and report the results of the data (mean test results

with standard deviation, regression models, statistical power etc., and/or other appropriate

determinants of the results) to evaluate the strength of the association between ADHD and

mathematical ability.

The maximum quality score for longitudinal studies was 11, while for cross-sectional studies it was 9. The longitudinal studies with a score of 9 or above were rated as 'high' quality, those with a score of 5 to 8 were rated as 'medium' quality and those with 0 to 4 were rated as 'low quality'. Similarly, cross-sectional studies with a score of 7 or above were rated as 'high' quality, those with 4 to 6 points were rated as 'medium' quality and those with 0 to 3 points were rated as 'low quality'. It is important to note that these cut-off points were agreed in advance by the authors to assess the degree of quality and bias.

***Genetic and Environmental correlations****.*

Genetic correlation (r_a_), indexes the extent to which genetic factors influencing mathematics also affect ADHD. If there were no overlapping genetic influences, r_a_ would be 0; conversely, if all genetic influences contributing to individuals' similarity in mathematics would also contribute to similarity in ADHD, r_a_ would be 1. Similarly, shared environmental correlation (r_c_) indexes common environments influencing mathematics and ADHD contributing to similarity in both traits. Non-shared environmental correlation (r_e_) indexes environmental effects, non-shared between family members which influence both mathematics and ADHD.

Genetic and environmental associations can be expressed in terms of unstandardised parameters (covariances) rather than standardised parameters (correlations).
